# Single-Cell Technologies to Understand the Mechanisms of Cellular Adaptation in Chemostats

**DOI:** 10.3389/fbioe.2020.579841

**Published:** 2020-12-18

**Authors:** Naia Risager Wright, Nanna Petersen Rønnest, Nikolaus Sonnenschein

**Affiliations:** ^1^Novo Nordisk A/S, Bagsvaerd, Denmark; ^2^Department of Biotechnology and Biomedicine, Technical University of Denmark, Kongens Lyngby, Denmark

**Keywords:** chemostat cultivation, continuous biomanufacturing, adaptation, population heterogeneity, microbes, single-cell technologies

## Abstract

There is a growing interest in continuous manufacturing within the bioprocessing community. In this context, the chemostat process is an important unit operation. The current application of chemostat processes in industry is limited although many high yielding processes are reported in literature. In order to reach the full potential of the chemostat in continuous manufacture, the output should be constant. However, adaptation is often observed resulting in changed productivities over time. The observed adaptation can be coupled to the selective pressure of the nutrient-limited environment in the chemostat. We argue that population heterogeneity should be taken into account when studying adaptation in the chemostat. We propose to investigate adaptation at the single-cell level and discuss the potential of different single-cell technologies, which could be used to increase the understanding of the phenomena. Currently, none of the discussed single-cell technologies fulfill all our criteria but in combination they may reveal important information, which can be used to understand and potentially control the adaptation.

## Introduction

Today, production of biological products is primarily based on batch operations where each unit operation is completed in sequence. The transition from these constitutive batch processes to continuous manufacture in which the product moves directly from one unit operation to the next, has been of growing interest within the bioprocessing community in recent years (Farid, 2019). Several benefits of moving to continuous processes can be listed due to the possibility of keeping production organisms in high producing states for longer time. These include a reduction in equipment costs, increased productivity, greater flexibility, and improved product quality (Zydney, 2016).

Continuous cell culture technologies have existed for several decades and include among others chemostat processes (Monod, 1950; Novick and Szilard, 1950). In a chemostat, the cells in the bioreactor are kept in a steady-state growth environment by a continuous addition of medium with one or more cell-density-limiting nutrients and simultaneous removal of spent culture medium at a defined rate (Peebo and Neubauer, 2018). Ideally, the chemostat should operate at a true steady state with a constant productivity. Although, the chemostat establishes a well-controlled and constant environment for production processes, it imposes selective pressure on cells, which may result in cellular adaptation. These alterations can affect productivity and the output of the cultivation. It is therefore important to understand the mechanisms behind the adaptation in order to control them and realize the full potential of chemostats in industrial production.

This article focuses on chemostat cultivations of microbes, the reported adaptation, and discuss how development of continuous biomanufacturing and chemostat processes can benefit from single-cell technologies.

## Adaptation in the Chemostat

**FIGURE 1:**
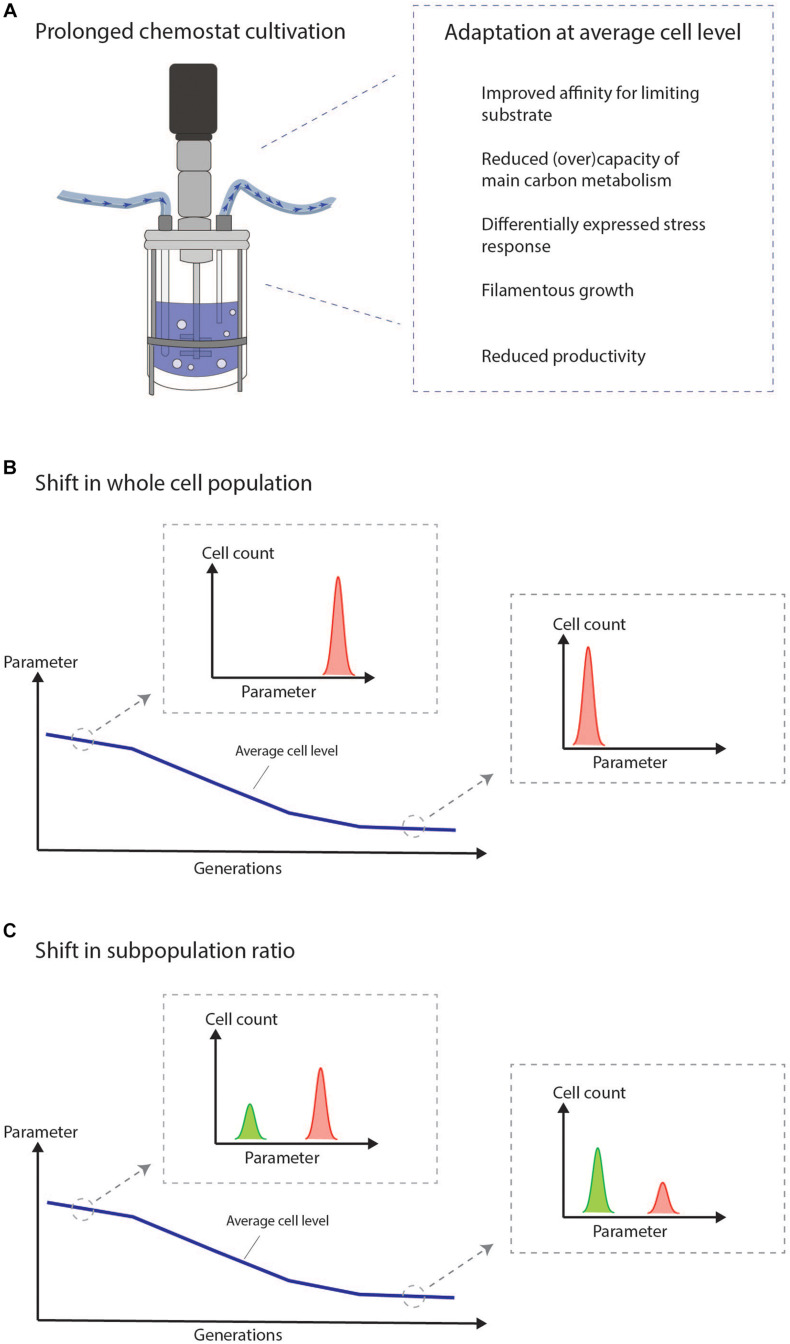
The chemostat imposes a selective pressure on the cultured cells, which drives cellular adaptation. We suggest to intensify the efforts on combining the study of adaptation at the average cell level with the current knowledge of population heterogeneity in chemostats to study the mechanisms of adaptation at the single cell level. **(A)** General trends observed at average cell level during prolonged adaptation in chemostat cultivation of microbes. **(B)** Illustration of adaptation measured in the bulk. The figure illustrates how it may look at the single-cell level if the adaptation is a result of a shift in the whole cell population. **(C)** Illustration of adaptation measured in the bulk. The figure illustrates how it may look at the single-cell level if the adaptation is a result of a shift in subpopulation ratios.

The chemostat imposes a steady nutrient limited environment forcing cells to grow at a constant growth rate. These conditions result in an ongoing selective pressure driving the adaptation of cells with growth advantages. Cells which are not able to adapt will be washed out. Adaptive processes in chemostats are illustrated in several studies for many different microorganisms at both the RNA, protein, metabolite, and morphological level (Adams et al., 1985; Ferea et al., 1999; Wick et al., 2001; Robin et al., 2003; Jansen et al., 2004, 2005; Mashego et al., 2005; Franchini and Egli, 2006; Wu et al., 2006; Douma et al., 2011; Paulová et al., 2012; Wang et al., 2018). The adaptation covers the differential expression of thousands of genes and proteins, but some general trends, which confer fitness in a nutrient limited environment, can be extracted (Figure 1A). This includes an improved affinity for the limiting substrate (Wick et al., 2001; Jansen et al., 2005). Any adaptation which increases the specific growth rate under low external concentrations of the limiting nutrient will improve the competitiveness of the cell compared to non-adapted cells (Jansen et al., 2005). Moreover, decreased (over)capacity of the main carbon metabolism including the glycolysis and TCA cycle is observed and has been suggested as a way to get an energetical advantage (Mashego et al., 2005). Cellular stress-responses are in many cases also differentially expressed between early and late cultivation stages including proteins involved in heat shock, oxidative stress, and damage resistance (Jansen et al., 2005; Franchini and Egli, 2006; Wright et al., 2020). Morphological changes toward filamentous and pseudo-hyphal growth are known effects of chemostat growth (Brown and Hough, 1965; Adams et al., 1985; Rebnegger et al., 2014; Rai et al., 2019) and also a known adaptive response to nutrient poor environments (Gimeno et al., 1992). The productivity of industrially relevant strains often decreases over time during chemostat growth (Douma et al., 2011; Paulová et al., 2012; Kazemi Seresht et al., 2013; Wright et al., 2016, 2020). A reduced productivity can be construed as a clear growth advantage over cells that are not able to reduce the burden of heterologous production.

Stochastic, regulatory, epigenetic, and mutational changes can contribute to increased fitness and adaptation is therefore a comprehensive process (Ryall et al., 2012). The underlying functional mechanisms of the adaptive processes in chemostats often remain unknown but many studies couple changed phenotypes to specific genetic mutations (Brown et al., 1998; Dunham et al., 2002; Wenger et al., 2011; Kvitek and Sherlock, 2013; Gresham and Hong, 2015; Hope et al., 2017). Hope et al. (2017) related morphological changes in *S. cerevisiae* after hundreds of generations to genetic mutations in known flocculation genes such as the cell wall protein FLO1. Clones with mutations in nutrient signaling and regulation of glucose transport have also been isolated from *S. cerevisiae* evolved for more than 200 generations in glucose-limited conditions (Wenger et al., 2011). This illustrates that some of the observed adaptive phenomena can be related to genetic alterations. Whether and when a given mutation will dominate a culture depends on the relative fitness of the mutant compared to other clones in the population (Gresham and Hong, 2015). For industrial strains, studies report reproducible adaptive changes in transcriptome, proteome, and heterologous product already after 22 generations of chemostat growth (Douma et al., 2011; Kazemi Seresht et al., 2013; Wright et al., 2020). The observed changes cannot always be coupled to genetic instability (Douma et al., 2011) and may therefore be related to other adaptive mechanisms, e.g., epigenetics.

Population heterogeneity is a cellular response to nutrient limitation and is reported for chemostat growth (Lieder et al., 2014; Kopf et al., 2015; Schreiber et al., 2016). Here we refer to population heterogeneity as the phenotypic diversity occurring between genetically identical individuals (Davis and Isberg, 2016). Nikolic et al. (2017) showed cell-to-cell variations in gene expression and substrate specialization for *E. coli* growing simultaneously on glucose and arabinose under chemostat conditions. Population heterogeneity with respect to growth and cell robustness was observed in glucose-limited chemostats of both *S. cerevisiae*, *E. coli*, and *P. putida* (Carlquist et al., 2012; Heins et al., 2019; Sassi et al., 2019) and also *Arthrobacter* evolves subpopulations with respect to nucleic acid content and metabolic activity (Kundu et al., 2020). The subpopulation ratios reported, strongly depend on the cultured strains, the cultivation conditions and the parameters analyzed. Ratios up to 1:2 between non-growing and growing subpopulations are reported (Kundu et al., 2020).

When bioprocesses are scaled up to manufacturing scale, the cells will often be exposed to a heterogeneous environment, for example, gradients in substrate and oxygen (Oosterhuis and Kossen, 1984; Larsson and Enfors, 1988). Fluctuations in the extracellular environment affect metabolism including product yield and by-product formation (George et al., 1993, 1998; Neubauer et al., 1995; Bylund et al., 1998, 1999, 2000; Lin and Neubauer, 2000; Enfors et al., 2001; Sandoval-Basurto et al., 2005). The gradients can also influence population heterogeneity. Differences in transcription levels between cells located in different zones of reactors have been found (Schweder et al., 1999; Lara et al., 2006). Schweder et al. (1999) measured different mRNA levels of stress genes between cells taken from the top and bottom of a production reactor. Nonetheless, other studies have shown that the heterogeneous environment can also result in a more homogeneous population for example when measured by viability and membrane damage (Hewitt et al., 2000, 2007; Han et al., 2013; Brognaux et al., 2014).

Population heterogeneity can therefore arise due to external influences such as gradients in manufacturing scale and nutrient limitation. It can also originate from intracellular events not influenced by the environment such as stochastic gene expression, e.g., random variations in the abundance of intracellular molecules with important regulatory functions (Elowitz et al., 2002; Blake et al., 2003). The population heterogeneity can have important functional consequences, which is beneficial for the entire population. It has been suggested that heterogeneity emerges as a consequence of metabolic cooperation between cells (Campbell et al., 2016) and that the population as a whole benefits from division of labor between individuals (Reuven and Eldar, 2011; Ackermann, 2015). We speculate that this can also occur in chemostats. Bet-hedging is another strategy resulting in phenotypic heterogeneity and can be seen as a way to cope with unforeseen conditions in fluctuating environments (Thattai and van Oudenaarden, 2004; Kussell and Leibler, 2005). Acar et al. (2008) suggested that isogenic populations can improve fitness by optimizing the phenotypic diversity to an ideal fraction. As a recent example Kundu et al. (2020) showed how cells in a chemostat divide into growing and non-growing subpopulations and propose that the isogenic population in this way improves its fitness to sudden increases in nutrient concentrations. Due to the selective nature of the chemostat, this strategy requires that cells with the less beneficial growth advantage continuously emerge, as they would otherwise be washed out (Kundu et al., 2020). Alternative mechanisms causing phenotypic heterogeneity can be related to aging and the asymmetrical division of exponentially growing cells. Recently, Li Y. et al. (2020) showed that genetically identical yeast cells age at different rates and toward different phenotypes in a constant glucose-limited environment, for example.

## Discussion of Single-Cell Technologies for the Study of Adaptation in Chemostats

It is essential to understand the functional molecular basis of adaptation in prolonged chemostats in order to utilize the full potential of the chemostat process in continuous biomanufacturing. We suggest to intensify the efforts on combining the study of adaptation at the average cell level with the current knowledge of population heterogeneity in chemostat cultivations to study mechanisms of adaption at the single-cell level. This could reveal important differences between subpopulations potentially hidden in bulk measurements (Figures 1B,C). For this endeavor, single-cell technologies are needed.

Traditionally, flow cytometry has been used to address heterogeneity in bioprocesses including chemostats (Hewitt et al., 1998, 1999; Delvigne et al., 2015; Heins et al., 2019; Vees et al., 2020). Populations differentiated by structural or physiological cell parameters can be revealed based on optical signals from, e.g., staining dyes or biosensors. Online flow cytometers exists and can be applied for regulation of bioreactors (Sassi et al., 2019). If the adaptation observed in chemostats is grounded in population heterogeneity, real-time monitoring of heterogeneity can potentially be used to control adaptation. However, more knowledge about how to control the processes are needed. Fluorescence-activated cell sorting in combination with proteomics or transcriptomics allow for the sorting of cells into subpopulations, which can afterward be analyzed by subpopulation omics (Achilles et al., 2007; Jehmlich et al., 2010; Jahn et al., 2013; Lieder et al., 2014). This method can be used to gain knowledge about changes in gene and protein expression leading to the development of subpopulations (Jahn et al., 2013). The method is limited by the time it takes to obtain enough cells to detect sufficient amounts of proteins or transcripts for the omics characterization.

Microfluidic single-cell cultivation systems enable time-resolved analysis of individual cells in accurately controlled environments by application of, e.g., online fluorescent readouts or phase contrast images. These systems are typically used to study cell division, morphology, aging, or gene expression (Elowitz and Leibler, 2000; Wang et al., 2010; Ullman et al., 2013; Grünberger et al., 2015; Li Y. et al., 2020). Contrary to studies in bioreactors, it is possible to follow phenotypic development and regulation of isolated cells with spatiotemporal resolution and to distinguish contributions from intrinsic stochastic processes and environmental factors (Weibel et al., 2007; Dusny and Schmid, 2015). On this basis, the systems can reveal fundamental insight into cellular regulation strategies to nutrient-limited conditions (Lindemann et al., 2019). Several microfluidic cultivation concepts exist where cells are trapped by different physical principles. This includes systems with contactless trapping of single cells by a non-uniform electric field (Kortmann et al., 2009; Fritzsch et al., 2013) and mechanical trapping of cells in chambers (Wang et al., 2010; Long et al., 2013). 1D chamber systems can accurately reproduce the dynamic nutrient variations observed by cells in a large-scale production reactor (Ho et al., 2019). We find the contactless cultivation systems most interesting for the study of adaptation to nutrient limited growth. In these systems cell-to-surface and cell-to-cell interactions are avoided (Fritzsch et al., 2013). However, cell-to-cell interactions may play important roles in bioreactors. Cross-scale studies have, e.g., revealed differences in growth rates of *C. glutamicum* due to density differences (Grünberger et al., 2013). 2D-chamber systems exist where cell-to-cell interactions can be examined (Burmeister et al., 2019). However, growth is restricted to two spatial dimensions in the 2D systems and gradients of nutrients and excreted metabolites can occur (Ho et al., 2019). Droplet microfluidics is another example of single-cell cultivation systems that enable high-throughput studies of adaptation. Jakiela et al. (2013) developed a micro-droplet chemostat to study bacterial growth and adaptation. However, it is hard to control the environment in the droplets (Schmitz et al., 2019). Recent examples show how microfluidic cultivation systems can be coupled to mass spectrometry (MS) for label-free analysis of extracellular proteins or metabolites (Dusny et al., 2019; Haidas et al., 2020; Schirmer et al., 2020). These setups are promising as they expand the window of molecules, which can be analyzed in microfluidic cultivation systems. However, the cultivation medium needs to satisfy the MS used for analysis (Schirmer et al., 2020). Therefore, it is hard to precisely match the bioreactor conditions in these setups.

Single-cell omics technologies covering single-cell proteomics, single-cell transcriptomic (scRNA-seq), single-cell genomics (SCG), and single-cell metabolomics are successfully applied to mammalian cells in areas such as health and disease (Baslan and Hicks, 2017; Stubbington et al., 2017). We envision a transfer of this success to microbial bioprocesses enabling the study of adaption in chemostats on all hierarchical levels by a systems biology approach (Joyce and Palsson, 2006). Methods for analysis of unicellular microorganisms lag behind mainly due to the small size of the microorganisms, the low number of molecules per cell, and their resistant cell walls (Saint et al., 2019). scRNA-seq and SCG are the most evolved methods, mainly because DNA and mRNA signals can be amplified. SCG has been used to study genomic heterogeneity in both monocultures and microbial communities by analyzing thousands of single cells (de Bourcy et al., 2014; Hosokawa et al., 2017; Lan et al., 2017). Recent promising studies have applied scRNA-seq for investigation of microbes (Gasch et al., 2017; Kuchina et al., 2019; Saint et al., 2019; Geoghegan et al., 2020; Jackson et al., 2020; Jariani et al., 2020). The newest technologies identified more than half of the transcriptome of yeast per cell for 285 individuals (Nadal-Ribelles et al., 2019). Methods for label-free, single-cell analysis of proteins, and metabolites from microbes are limited. Single-cell MS has been used to measure in the region of 25 intracellular metabolites from up to a thousand single cells from microbial cultures (Ibanez et al., 2013; Walker et al., 2013; Krismer et al., 2017; Li Z. et al., 2020). To our knowledge, only few methods for unlabeled single-cell proteomics of microorganisms exist in literature. Armbrecht et al. (2019) published a single-cell proteomics method for mammalian cells and claimed that it also works for microbes. Sensitivity for detection, technical noise and the wide range of expression levels have been highlighted as main challenges for MS techniques (Zhang and Vertes, 2018). To speed up the progress toward suitable single-cell omics methods one could focus the development on more efficient sample preparation methods, e.g., based on microfluidics or droplets that lead to less loss of material due to adsorption to plastic etc. (Dou et al., 2019). Moreover, to increase the coverage of the methods, we see computational approaches, which impute missing data, as important tools (Li and Li, 2018; Peng et al., 2019).

**FIGURE 2:**
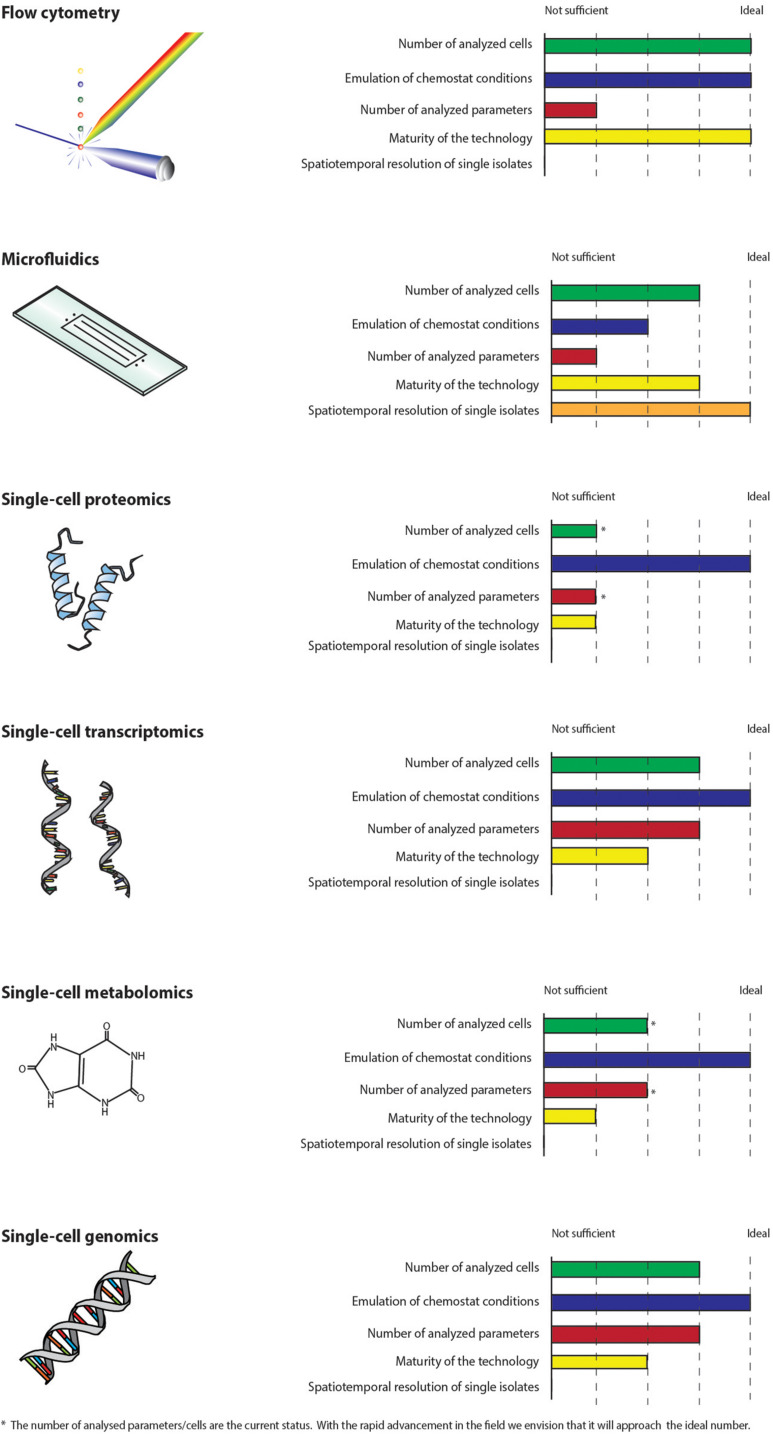
Single-cell technologies are compared on a scale from not sufficient to ideal with respect to how suited they are for the study of adaptation in chemostats. The parameters used for the comparison are: the number of analyzed cells, how well the technologies emulate chemostat conditions, the number of parameters which can be analyzed, how mature the technologies are and whether the technologies can be used to study individual cells by precise spatial and temporal control. None of the technologies *fulfill* all these criteria at the moment.

The ideal technology for the study of single-cell adaptation in chemostats should be high-throughput with respect to the number of phenotypic parameters that can be analyzed and in terms of the number of cells investigated in order to be statistically significant for the high cell density cultures applied in industry. Moreover, the cells should be analyzed in an environment, which emulates chemostat conditions and the analysis should not interfere with the studied mechanisms. Ideally, the technology should enable a spatiotemporal resolution of individual cells. None of the technologies described in this perspective article fulfill all these criteria (Figure 2), but together they can be used to reveal the underlying mechanisms thus enabling control of adaptation in chemostats, e.g., by bioprocess control strategies or metabolic engineering. Flow cytometry is well established, high-throughput in terms of the number of cells analyzed and has already proven its worth for the study of chemostats (Hewitt et al., 1998; Sassi et al., 2019). The online versions can be coupled directly to bioreactors making it possible to perform the analysis in a relevant environment. Microfluidic cultivation systems are the only technology, which enables the study of individual cells by precise spatial and temporal control. However, the systems have to emulate bioprocess conditions. The number of available biosensors and reporter strains are rapidly increasing. Recently, a biosensor for the measure of glycolytic flux in yeast was developed (Monteiro et al., 2019). Combined with flow cytometry and/or microfluidics, the increase in reporter strains and biomarkers will enable the study of new phenomena and mechanisms on the single-cell level. Technologies, which rely on fluorescent readouts, are restricted by the number of dyes, which can be applied simultaneously. Moreover, genetic modifications of host strains for incorporation of biosensors can be work intensive and might interfere with the metabolism of the host. Due to the cost of large-scale production reactors, potential GMP and safety regulations, we find it hard to imagine that reporter strains can be used to investigate adaptation in actual production scale. Single-cell metabolomics and proteomics are still not suitable for the analysis of microbial bioprocesses. SCG technologies are more matured but will only reveal mutational mechanisms. scRNA-seq has advanced rapidly but can be costly and labor intensive (Nadal-Ribelles et al., 2019), which may explain the limited application in microbial bioprocesses. Further development of the technologies are therefore needed. Adaptation in chemostats affects both the genome, transcriptome, metabolome, and proteome (Dunham et al., 2002; Kazemi Seresht et al., 2013; Wright et al., 2020). We envision the application of single-cell omics for the holistic study of the adaptive mechanisms, as the omics technologies have the potential to measure large amounts of parameters at all regulatory levels. Therefore, if the rapid advancements of the technologies continue, single-cell omics can become important supplements to flow cytometry and microfluidic cultivation systems.

## Data Availability Statement

The original contributions presented in the study are included in the article/supplementary material, further inquiries can be directed to the corresponding author.

## Author Contributions

NW and NS conceived the idea for the article. NW reviewed the literature and wrote the manuscript. NS and NR critically commented on the manuscript and contributed their perspectives. All authors contributed to the article and approved the submitted version.

## Conflict of Interest

NW and NR were employed by the company Novo Nordisk A/S. The remaining author declares that the research was conducted in the absence of any commercial or financial relationships that could be construed as a potential conflict of interest.
